# Red Yeast Improves the Potential Safe Utilization of Solid Waste (Phosphogypsum and Titanogypsum) Through Bioleaching

**DOI:** 10.3389/fbioe.2021.777957

**Published:** 2021-12-31

**Authors:** Haoming Chen, Yuqi Lu, Chaonan Zhang, Fangfang Min, Zongli Huo

**Affiliations:** ^1^ School of Environmental and Biological Engineering, Nanjing University of Science and Technology, Nanjing, China; ^2^ Jiangsu Provincial Center for Disease Control and Prevention, Nanjing, China

**Keywords:** red yeast, bioleaching, biomineralization, extracellular polymeric substance adsorption, phosphogypsum, titanium gypsum, resource and safe utilization

## Abstract

Phosphogypsum (PG) and titanium gypsum (TG), as a by-product (solid waste) in phosphate fertilizer and titanium dioxide industry, are causing serious environmental hazards. The resource/harmless application of PG and TG is the development trend in the future. The biological function of red yeast (Rho: *Rhodotorula mucilaginosa*) can effectively reduce the concentration of pollutants in the environment and has the potential of biological flotation/purification of mineral solid waste. In this study, the bioremediation mechanism and safe utilization efficiency of Rho for different contents of PG and TG were explored by using its biological flotation function. The X-ray fluorescence spectrometry (XRF) results showed that F was the main toxic element in PG and TG, and Pb and Cd did not reach the detection limit. The processing capacity of Rho for PG (>10 g/ml) is higher than that of TG (<5 g/ml). After bioleaching by Rho, the proportion of F in PG and TG solid decreased by 61.45–63.79% and 49.45–59.19%, respectively. The results of three-dimensional fluorescence, extracellular polymeric substance (EPS) extraction, X-ray diffraction (XRD), and scanning electron microscopy (SEM) confirmed that Rho could accelerate the release of harmful elements (F) in PG and TG. SEM showed that Rho cells and secretions adhered and wrapped on PG/TG, causing PG/TG decomposition and fragmentation. In addition, the adsorption of EPS and the formation of Ca_5_(PO_4_)_3_F are two main ways for Rho to remove F. Furthermore, under the condition of high concentration bioleaching, Rho can accelerate the release and utilization of P in PG, which is not only for the re-precipitation of Ca_5_(PO_4_)_3_F but also conducive to the reproduction and utilization of microorganisms. Meanwhile, the purification/safe reuse of PG by Rho is easier than that of TG. Therefore, the toxicity of PG and TG bioleaching by Rho can be greatly reduced, suggesting the huge potential of Rho in soil improvement and remediation.

## Highlights

• The biological resource utilization of phosphogypsum (PG) and titanium gypsum (TG) by Rho was carried out for the first time;
• Rho can reduce the harm of PG and TG by producing fluorocalcium phosphate;
• Rho can adsorb F in PG and TG through extracellular polymer;
• Rho can accelerate the release of P in PG/TG, which is conducive to passivation and fixation of harmful elements (F-phosphorus minerals).
• The F stress of TG is stronger than that of PG, and its biological purification is more difficult.

## Introduction

Phosphogypsum (PG) is a complex solid waste produced during the production of phosphate fertilizer and phosphoric acid from the raw phosphate ore using a wet-process treatment (Papastefanou et al., 2006). The main constituents of PG are anhydrous calcium sulfate and massive impurities including phosphate, fluoride, and heavy metals (Hao et al., 2005; Rashad, 2017; Li and Zhang, 2020; Romero-Hermida et al., 2020). Titanium gypsum (TG) is an industrial by-product from the titanium dioxide industry. Like PG, it consists mainly of crystalline gypsum (CaSO_4_ž2H_2_O) (Gazquez et al., 2013). Since 2018, China’s annual output of PG and TG is about 78 million tons and 20 million tons, respectively, but the rate of resource comprehensive utilization is less than 40%, and even the domestic accumulation of PG has exceeded 500 million tons (Li et al., 2020; Xie et al., 2021). In addition, due to the complex composition of PG and TG (such as, Pb, F, P, Fe, and Si), discarding/stacking them in large quantities will lead to soil hardening, eutrophication, heavy metal pollution, and other serious environmental pollution problems (Li et al., 2020; Marchi et al., 2020; Costa et al., 2021).

PG and TG are often used to produce gypsum board, high-end ceramics, cement retarder, functional filler, and decorative materials (Jiang et al., 2016; Harrou et al., 2020; Zhai et al., 2020). However, these utilization modes are limited by the complex production process and finite output, and the secondary pollutants extracted are difficult to be treated, which will also cause environmental problems (Li et al., 2020; Romero-Hermida et al., 2020). Therefore, it is necessary to choose a new application way to improve the resource utilization efficiency and security of PG and TG. Soil quality problem has always been the hotspot of environmental research, and one of the existing methods is nutrient supplement, and structural improvement/restoration was performed by using fertilizer, mineral, biochar, microorganism, or other ways (Zhao et al., 2017; Chen H. et al., 2018; Chen et al., 2019; Gao et al., 2020; Liu J et al., 2021). However, the high cost of the soil remediation agent limits its application and popularization in soil improvement. Using harmless solid waste to improve or repair soil will be the focus of future research because solid waste has low cost and is easy to be supported by the state. Studies have shown that the use of marble dust can improve soil properties (Ural et al., 2014). Ca^2+^ from calcium sulfate can not only precipitate or adsorb soluble P in soil but also improve soil structural characteristics (such as aggregate stability), so as to control soil erosion (Pietola, 2008; Chen et al., 2016). PG and TG are rich in CaSO_4_, which provides a new idea for soil improvement. Studies have shown that 1.7 million tons of PG is used as an agricultural soil conditioner in Brazil every year (Costa et al., 2021). In addition, PG and TG contain enough nutrient elements such as P, K, and S, which provides necessary conditions for crop growth (Peacock and Rimmer, 2000). Meanwhile, TG has a high flocculation rate in the soil, so it can prevent soil loss to a certain extent (Fauziah et al., 1996).

The detrimental contained in PG and TG often hinders their application in the environment. For example, the Pb element in the PG and TG is easy to migrate in the environment and does harm to human health (Lütke et al., 2020). The fluorine ions can enter the body through the food chain and cause skeletal fluorosis and organ disease (Lv et al., 2021). Therefore, the pretreatment method must be adopted to reduce the environmental harm of PG and TG before their use. Heavy metal and fluorine are mostly separated from compounds by using chemical methods (hydrofluoric acid, NaOH, *etc*.) and then treated (precipitation method, adsorption method, ion exchange method, *etc*.) (Greenland, 1962; Larsen, et al., 2019; Chen et al., 2019; Chen et al., 2020). Compared with physical/chemical purification methods, bioremediation/bioleaching is one of the most potential environmental pollution remediation and pollutant extraction technologies (Wang et al., 2013; Pushkar et al., 2021). It can reduce the harmfulness and migration of multiple pollutants in the environment through microbial metabolism and secretions. For example, yeast can reduce Pb (650 mg/L) and Cd (5–12 mg/L) in the environment (Salinas et al., 2000; Hietala et al., 2006; Li et al., 2019). Fungi can separate and adsorb fluorine in fluorine-containing minerals by secreting organic acids (Shao et al., 2021). The EPS of microorganism can adsorb a variety of pollutants, such as Pb, Cd, and Cu (Sheng et al., 2012; Wang et al., 2020; Pagliaccia et al., 2021; Ye et al., 2021). Dry baker’s yeast cells can mineralize and fix Cu by releasing P at the long term (Ojima et al., 2020). Besides, microorganisms can accelerate the release of nutrient elements in soil remediation agents and improve the remediation effect of remediation agents (Chen et al., 2020; Shao et al., 2021). However, improving the safety and resource utilization efficiency of solid waste (PG and TG) by bioremediation has not been studied.

In this study, bioremediation was performed on different amounts of PG and TG by using the biological function of red yeast (leaching and purification). It was hypothesized that the organic secretion (EPS and acid secretion) of red yeast could effectively release and adsorb the harmful elements in PG and TG. The X-ray fluorescence spectrometry (XRF), inductively coupled plasma spectrometer (ICP-OES), and ion chromatography were used to determine Pb, P, F, and other elements. Then X-ray diffraction (XRD), attenuated total reflection infrared spectroscopy (ATR-IR), and scanning electron microscopy (SEM/EDS) were used to analyze the safe utilization effect of red yeast on PG and TG, and the extracellular polymer gradient centrifugation technique and three-dimensional fluorescence (3D-EEM) were used to further explore the safe utilization mechanism of red yeast on TG and PG.

## Materials and Methods

### Materials

**FIGURE 1:**
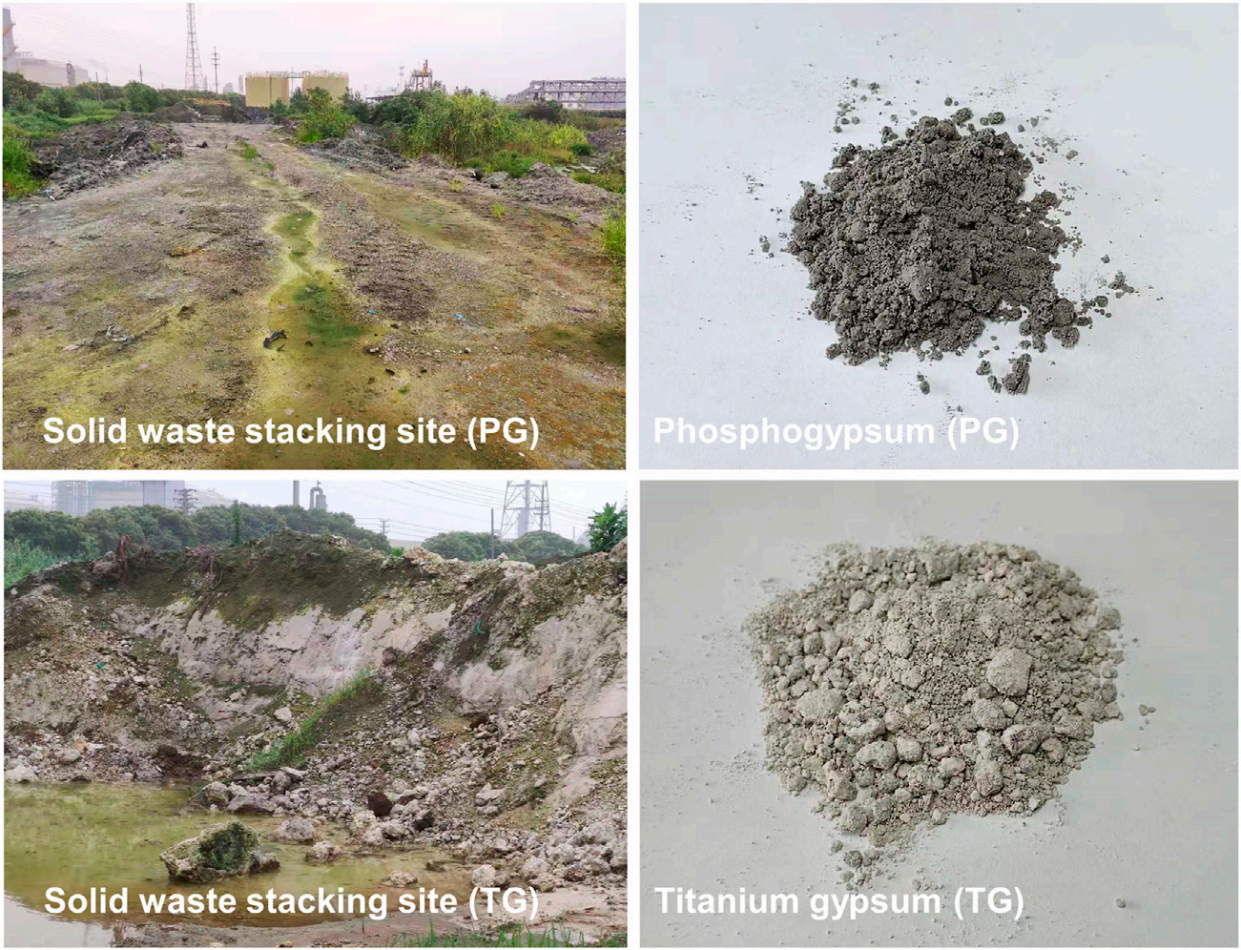
Solid waste dump site and two kinds of waste original morphology.

PG and TG samples used in this study were collected from a fertilizer and titanium dioxide production plant in Taizhou, Jiangsu Province, China (Figure 1). PG (dark gray) and TG (white) solids were ground in an agate mortar and dried (80°C, 24 h) after being screened through a 500 mesh sieve (<25 μm). Table 1 shows the chemical compositions of the raw materials analyzed using X-ray fluorescence spectrometry (XRF). Abundant P elements and some risky metal elements (F, Cu, Pb, and Zn) were found in PG and TG. Therefore, these risk elements were mainly analyzed during the experiment. The red yeast (Rho) strain was isolated from orchard rhizosphere soil (*Rhodotorula mucilaginosa*, CGMCC No.16597, Nanjing Agricultural University) (Li et al., 2019). Before the incubation, Rho was activated in the potato dextrose broth (PDB) medium at 28°C, 180 rpm for 48 h. This red yeast is characterized by its ability to secrete large amounts of EPS (Li et al., 2019; Jiang et al., 2020; Wang et al., 2020), which contributes to the adsorption of Pb, Cd, F, and Cu.

**TABLE 1:** Basic properties of phosphogypsum and titanium gypsum.

Phosphogypsum	Element name	Ca	S	Si	Al	P	Fe	F	K	Ba	Ti	Na	Mg	Sr	Mn	Cu	Ar	W	Hf	Pb	Rb	Zn
	wt%	15.500	7.920	2.030	0.319	0.246	0.388	0.348	0.288	0.102	0.066	0.076	0.053	0.060	0.009	0.003	0.003	0.002	0.002	0.002	0.001	0.001
		±0.002	±0.002	±0.001	±0.002	±0.001	±0.002	±0.003	±0.001	±0.001	±0.001	±0.001	±0.002	±0.001	±0.002	±0.001	±0.001	±0.001	±0.001	±0.001	±0.001	±0.001
**Titanium gypsum**	**Element name**	**Ca**	**F**	**S**	**Si**	**Al**	**Mg**	**Na**	**Fe**	**Cl**	**Sb**	**P**	**K**	**Ti**	**Sr**	**Er**	**Tm**	**Mn**	**Tb**	**Pb**	**Zn**	**As**
	wt%	30.490	5.440	0.955	0.697	0.718	0.471	0.424	0.371	0.406	0.295	0.044	0.0797	0.0355	0.0218	0.0190	0.0176	0.0114	0.0088	0.0079	0.0057	0.0052
			±0.001	±0.001	±0.002	±0.001	±0.001	±0.001	±0.001	±0.001	±0.001	±0.001	±0.002	±0.002	±0.001	±0.001	±0.001	±0.001	±0.001	±0.001	±0.001	±0.001	±0.001

### Bioleaching of PG/TG Using Rho Yeast and Activation for Adsorption Experiments

5 and 10 g PG or TG powder were put into the 150 ml yeast extract peptone dextrose (YPD) medium in a 250-ml conical flask, respectively, and marked as PG5, PG10, TG5, and TG10. The YPD culture medium is prepared with 20 g peptone, 10 g glucose, 20 g yeast powder, and 1,000 ml deionized water. The medium pH was adjusted to 7.0–7.4 with NaOH and HCl. All reagents are from Thermo Fisher Scientific, United States. Then 2 ml of Rho liquid was injected into the PG or TG medium after sterilization, and marked as PG5-Rho, PG10-Rho, TG5-Rho, and TG10-Rho. The culture medium (CK) and red yeast fluid (Rho) were treated as background solution and yeast treatment, respectively. All the treatments (ten groups) were oscillated for 5 days in a dark shaker at 180 rpm/min and 36°C, with three replicates. After sampling, the mixed solution was separated by solid–liquid separation (1,000 rpm, 30 min), and then the PG or TG, red yeast cells, and EPS in the suspension were separated using the gradient centrifugation and dialysis method (Wang et al., 2020). The total P, Pb^2+^, Cu^2+^, Zn^2+^, and F^−^ in the filtrate were determined by using ICP-OES and ion chromatography. Three-dimensional fluorescence (3D-EEM) was used to analyze the microbial secretions in the filtrate. The solid is divided into three parts: one part is calcined in a muffle furnace (1 g, 600°C, 4 h) to remove the organism on the solid, and the recovery quality is determined. A part of the solid was then immobilized with 2.5% glutaraldehyde (stored at 4°C) for the SEM analysis. The last part of the solid was analyzed using ATR-IR, XRD, and electrochemical techniques after drying (45°C).

### Extraction and Bioanalysis of Extracellular Polymeric Substance (EPS) in Extracellular Polymers

After 5 days of bioleaching experiments, the 60 ml filtrate was collected for EPS extraction (Wang et al., 2020). The filtrate was centrifuged at high speed (4°C, 12,000 g, 20 min), and then centrifuged again (4°C, 17,000 g, 20 min) to isolate cell and EPS. Then the supernatant after centrifugation was mixed with three times of absolute ethanol, and the supernatant was sealed for 48 h (4°C) to precipitate the extracellular secretion. The precipitate was transferred to the dialysis bag (3,500 molecules) and put into ultrapure water (replaced twice every 24 h) to remove small-molecule impurities such as ethanol. The extract was divided into two parts: one was freeze-dried for electrochemical determination. The other one was used for ICP and ion chromatography determination.

### Chemical and Microstructural Analyses

The elements (wt%) of solid were determined by using XRF (Rh 60 kV excitation source, ARL PERFORM’X, Thermo Fisher Scientific, United States). After drying (105°C, 48 h) and grinding (<74 μm), the sample is put into a muffle furnace for pyrolysis at 600°C (4 h), and then weighed 4.00 ± 0.01 g and pressed in a circular mold (32 mm) for XRF determination.

The concentration of soluble total phosphorus (HPO_4_^2−^, PO_4_^3−^, and H_2_PO_4_^−^), Zn^2+^, Cu^2^, and Pb^2+^ was determined using an inductively coupled plasma spectrometer (Agilent 710, ICP-OES). The F^−^ concentrations were analyzed using an ion chromatograph (ICS, Metrohm 940). The sample (liquid after solid–liquid separation) was filtered through a 0.22-μm filter membrane and then diluted 5 times for determination. The calibration of measured elements was performed by 0, 1, 5, 10, 25, and 50 mg/L standards (internal standard curve R = 0.999). The standard was provided by the National Center of Analysis and Testing for Nonferrous Metals and Electronic Materials, China.

The surface biological and mineralogical characteristics of the precipitates were determined by using a Nicolet iS5 infrared spectrometer (ATR-IR, 4 cm^−1^, 500–2000 cm^−1^, 16 times scanning; Thermo Fisher Scientific Inc., Madison, United States) and a Bruker D8 X-ray diffractometer (XRD, Pb-Ka; 40 kV; 40 mA; scanned from 5 to 65° at a speed of 0.02°/s; D8 Advance, Bruker AXS GMBH, Germany).

Biological and mineral morphologies of precipitates (glutaraldehyde fixation and spray gold for 5 min) were determined using scanning electron microscopy (SEM, Carl Zeiss SUPRA 55 system, acceleration voltage of 5–15 kV). Oxford Aztec X-Max 150 energy spectrometer (EDS) was used for the semi-quantitative analysis (acquisition time: 90 s) (Tian et al., 2019).

Three-dimensional excitation emission matrix fluorescence spectroscopy (3D-EEM, RF-6000: SHIMADZU, Shimadzu Corporation, Japan) was used to detect the organic matter generated during the operation with the spectral scanning speed of 6,000 nm/min (excitation (λ_Ex_) and emission wavelength (λ_Em_) = 200–450 nm, step width = 5 nm).

### Statistical Analysis

Three replicates were set for all treatments, and their average values and standard deviations were calculated. SPSS 22.0 was used for one-way analysis of variance (ANOVA) to evaluate the differences between different treatments (significance level *p* < 0.05). The infrared data analysis was carried out by using Thermo Scientific OMNIC software, and the XRD data analysis was carried out by JADE 6.0. Origin 8.0 was used for graphic processing.

## Results and Discussion

### Red Yeast Bioleaching Promotes the Release of Elements in PG and TG

**FIGURE 2:**
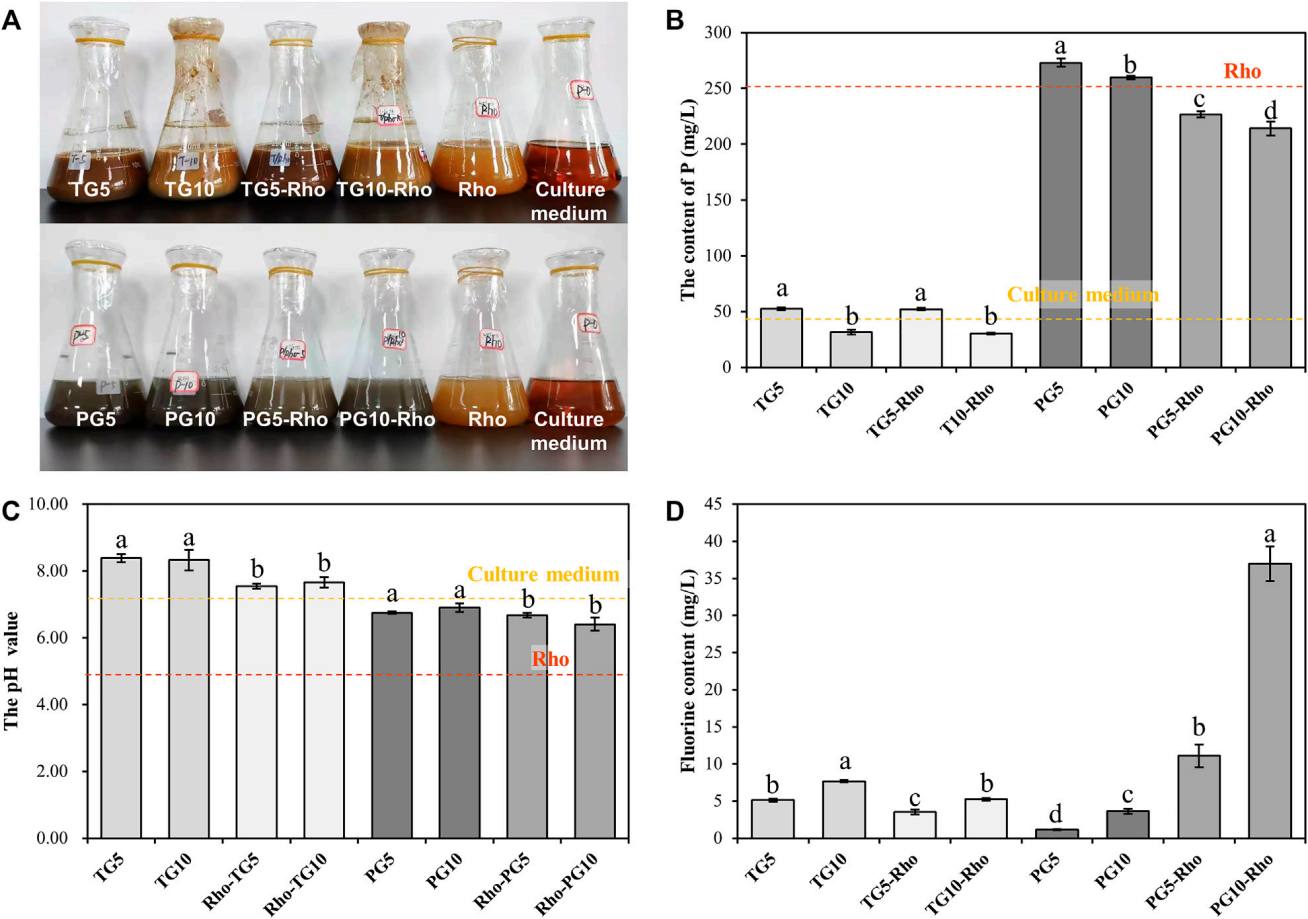
The solution turbidity of each treatment after reaction **(A)** and toal soluble P **(B)**, pH **(C)**, total F **(D)** content in solution. (TG: Titanium gypsum; PG: Phosphogypsum; Rho: Red yeast).

After the 5-day bioleaching experiment, the treatment with Rho addition (TG5/10-Rho and PG5/10-Rho) was significantly more turbid and lighter in color (Figure 2A), which showed that Rho produced obvious organisms and organic substances in PG and TG (such as cells and extracellular polymers substances). ICP-OES results showed that the content of Pb, Cd, Zn, and Cu elements was below the detection limit in PG or TG solution (<0.05 mg/L). The P content in the TG solution was significantly lower than that in the PG solution (Figure 2B). In addition, the higher the content of PG or TG addition, the lower the P content in the corresponding solution. When the addition amount of TG and PG increases from 5 to 10 g, the P content decreases by 39.9 and 48.4%, respectively. However, there was little difference between the P content in TG5 and that in the culture medium, while the P content in TG10 was lower than that in the culture medium. This phenomenon may be due to the complexation effect of mineral solid on the culture medium, and the more solid, the more P in the solution can be adsorbed. After adding TG and PG into pure water, it was found that both have P release, and the release of PG is higher than that of TG (Supplementary Figure S1), which proves that both TG and PG can provide guarantee for the utilization of phosphorus source of plants and microorganisms, and the difference between the two is mainly due to the lower P content of TG itself than PG (Table 1).

With the addition of Rho, there was no significant change in the content of P in TG, but the content of P in PG decreased significantly (Figure 2B). The P content of PG5-Rho was 17.0% lower than that of PG5, while the P content of PG10-Rho was 17.6% lower than that of PG10. Meanwhile, the content of P in the solution of Rho alone was ∼254 mg/L, which was lower than that in the treatment of PG5 and PG10. These results showed that Rho could survive normally in the 5–10 g PG treatment and utilize the P source of PG. However, there was no significant difference between the P content of TG5/10 and TG5/10-Rho (slightly decreased by 0.6%/4.1%), which showed that the growth of Rho was significantly inhibited in TG and reduced the utilization of P by Rho.

After Rho bioleaching, the pH of all treatments with Rho was lower than that without Rho (Figure 2C), which may be caused by the organic acids or enzymes produced by Rho (Nakayan, et al., 2013). The decrease of environmental pH is an important factor to improve mineral dissolution (Vemic et al., 2016; Wu et al., 2021). In addition, the better the yeast grows, the more obvious the pH decreases (the pH of PG5/10-Rho is lower than that of TG5/10-Rho). Stronger oxidizing power and corrosiveness are caused by the increase of H^+^, which is conducive to the release of elements in minerals and mineral purification.

The higher the addition of PG and TG, the greater the release of F (Figure 2D). After Rho bioleaching, the content of F in PG solution increased significantly. The content of F in PG5-Rho was 8.7 times higher than that in PG5, and the content of F in PG10-Rho was 9.1 times higher than that in PG10, which further shows that the leaching of F in PG is very limited by using the culture medium alone, and the leaching effect of Rho is obvious. This may be mainly due to the acid secretion produced by red yeast accelerating the dissolution of PG (Carlos et al., 2018), which is also confirmed by the decrease of pH in Rho treatments.

Contrary to the results of PG, the F content of TG5/10-Rho solution is lower than that of TG5/10 solution. Yeast can resist environmental harmful stress through its own cell proliferation and organism adsorption (Wang et al., 2020; Fan et al., 2021; Liu Y et al., 2021), so most of the F released in TG may be adsorbed by yeast cell or its secretions. However, TG contains a large amount of F; when yeast attaches to the surface of TG for decomposition, it will be directly stressed by F in TG, resulting in its inability to play the ecological function of dissolving mineral solids, which may also be the reason why the pH reduction of TG5/10-Rho is not obvious. Therefore, combined with biological properties (Figure 2A), it can be confirmed that the more Rho, the better the bioleaching effect.

### Safety and Utilization of Solid Elements After Bioleaching

**FIGURE 3:**
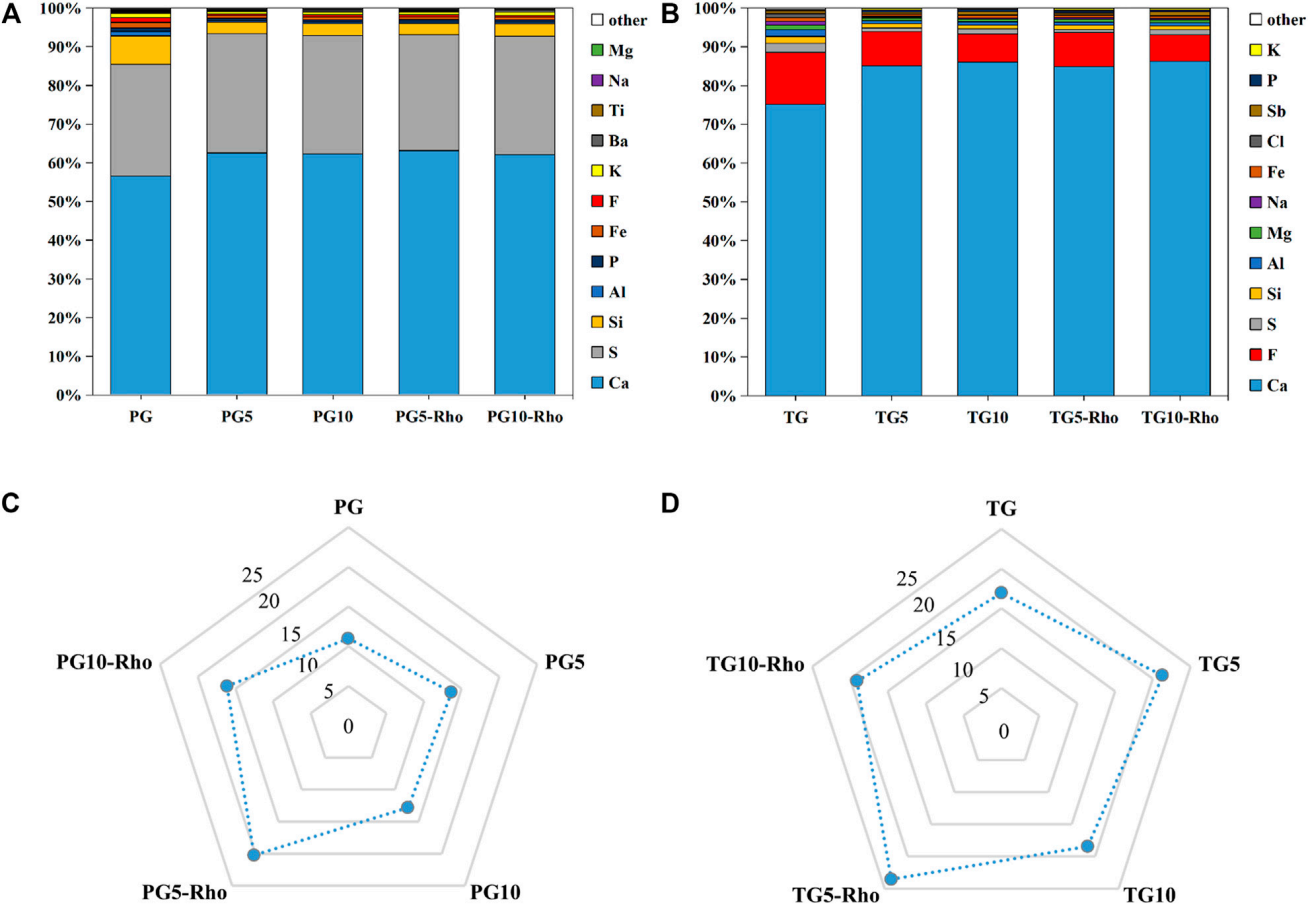
XRF analysis **(A,B)** and recycling rate **(C,D)** results of solid after bioleaching by Rho. (TG: titanium gypsum; PG: phosphogypsum; Rho: red yeast; unit is %).

XRF results show that Rho bioleaching can significantly reduce the impurities of TG and PG, and the proportion of Ca and S elements (TG and PG) is significantly increased, indicating that Rho has the effect of purifying solid waste. After bioleaching by Rho, the proportion of P in TG increased by 195.05–404.51% (Figure 3A), while that in PG decreased by 17.32–43.09% (Figure 3B), which confirmed that the P in PG was released by Rho, and PG has the potential of sustained release. Sufficient phosphorus content is the guarantee of soil quality improvement and crop growth (Ding et al., 2020; Tian et al., 2020). Therefore, the P release of PG by Rho makes PG have potential in soil improvement and agricultural application. In addition, the proportion of F in PG5/10-Rho and TG5/10-Rho solids were significantly lower than that in raw PG and raw TG by 61.45–63.79% and 49.45–59.19%, respectively (Figures 3A,B), which means that in the case of unit mass use, the contents of dangerous elements of TG and PG after bioleaching are greatly reduced and safer to use.

All treatments were dried and then pyrolyzed at high temperature to remove organic matter. The pyrolysis loss of raw PG and TG were 16.94 and 10.99%, respectively (Figures 3C,D). After mixing with the YPD medium, the pyrolysis loss of TG5/10 and PG5/10 were 18.45–21.29% and 12.80–13.65%, respectively, indicating that TG and PG have certain adsorption/complexation functions for organic substances in solution. This result also explains the phenomenon that P content in TG treatment solution is lower than that in the YPD medium. In addition, the higher the solid dosage, the lower the organic matter content. TG5 and PG5 are 15.39 and 6.64% higher than TG10 and PG10, respectively, which is because minerals can easily form agglomeration in the solution and do not fully contact with the solution (Shen et al., 2018).

Compared with raw TG and PG, Rho bioleaching TG and PG contain significantly more organic matter, and Rho improves the organic matter content in PG (25.78–48.28%) higher than TG (3.36–10.66%). Combined with the growth status of Rho, it can be found that the increase of organic matter in PG5-Rho may be due to Rho’s own extracellular secretion. As the most common organic secretion of yeast, extracellular secretion has the function of adsorbing harmful substances and heavy metal ions (Jiang et al., 2020), which is conducive to extracting harmful elements from solid waste and improving its safe utilization. In addition, organic matter can play a role in soil improvement, such as provide nutrient source for microorganisms and adjust soil moisture (Mack et al., 2014; Dhaliwal et al., 2019; Bonfante et al., 2020; Zhao et al., 2021). Some studies have found that local microorganisms can be used to cooperate with PG to biodetoxify PG, which further proves that microorganisms can improve the safe utilization efficiency of phosphogypsum (Sengupta and Dhal, 2021).

### Biomineralization Mechanism of Safe Utilization of PG and TG

**FIGURE 4:**
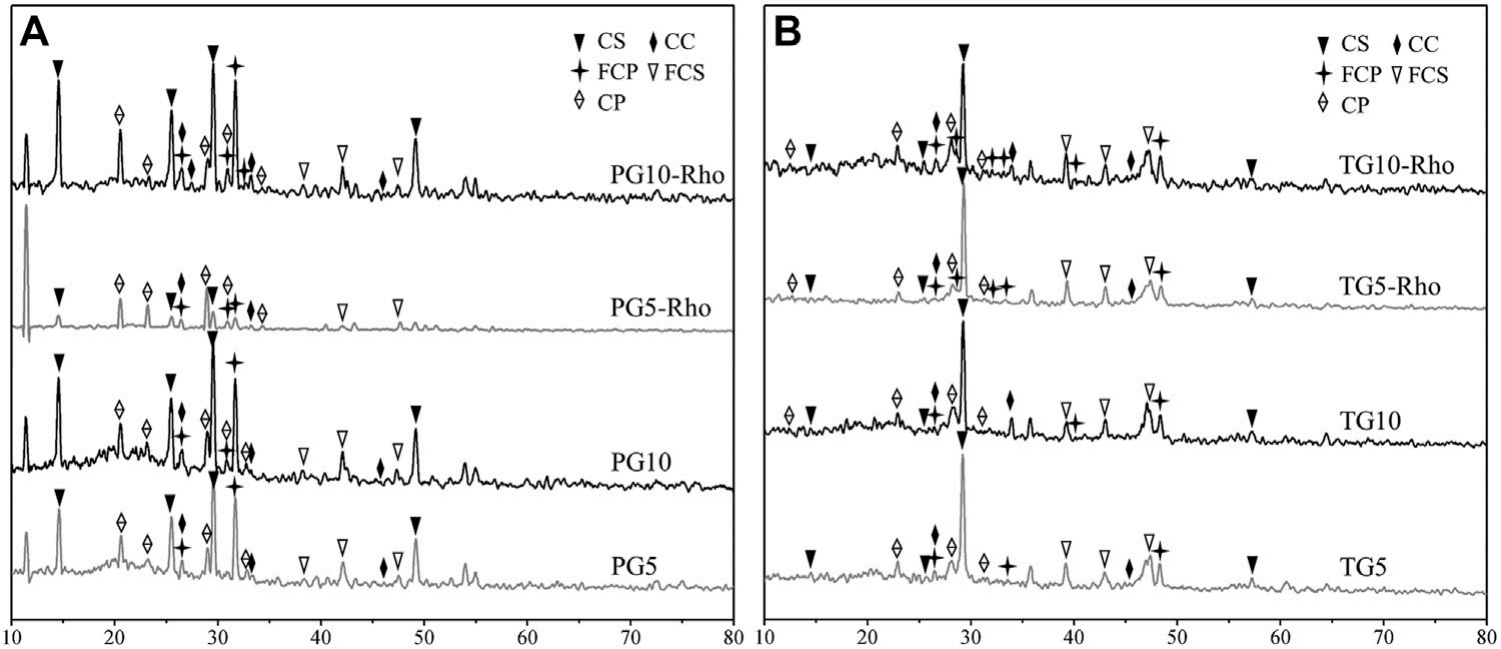
XRD analysis results of solid after bioleaching by Rho. {PG: phosphogypsum; TG: titanium gypsum; Rho: red yeast; CS: calcium sulfate (CaSO_4_); CP: calcium phosphate [Ca_3_(PO_4_)_2_]; CC: calcium carbonate (CaCO_3_); FCP: calcium fluorophosphates [Ca_5_(PO_4_)_3_F]}.

XRD results show that the main minerals in the sample are calcium sulfate (CaSO_4_), calcium phosphate [Ca_3_(PO_4_)_2_], calcium carbonate (CaCO_3_), calcium fluoride sulfate (Ca(SO_3_F)_2_), and calcium fluorophosphates [Ca_5_(PO_4_)_3_F] (Figure 4), which corresponded to the standard JCPDS card. The obvious peaks at 14.66°, 23.143°, 25.539°, 29.574°, 31.971°, and 32.864° indicate the presence of calcium sulfate (CaSO_4_). Combined with XRF results, it was found that calcium sulfate was the main mineral in TG and PG. In addition, another major mineral in TG is calcium fluoride sulfate (47.305°, 48.103°, 43.253°, 39.311°, 31.249°, 23.32°, and 14.390°), while that in PG is calcium phosphate (28.870°, 29.554°, and 31.871°).

After the bioleaching by Rho, a range of peaks appeared in TG and PG at 28.870°, 29.554°, and 31.871°, and 31.93°, 32.26°, 33.12°, 25.86°, 34.14°, and 40.04° were significantly enhanced (Figure 4). These peaks belong to the typical diffraction of Ca_5_(PO_4_)_3_F. Meanwhile, the signal of calcium phosphate was also significantly enhanced. Previous studies have confirmed that Rho can convert P release from TG and PG into PO_4_^3−^ (Carlos et al., 2018; Ojima et al., 2020), which is bound to F^−^- and Ca^2+^-shaped minerals. Because there are more fluorine elements in TG than in PG, the signal of fluorophosphate is also stronger, which is consistent with the XRF results. In addition, some carbonates were also found in TG (CaCO_3_: 26.212°, 27.215°, 29.4°, 33.127°, 36.175°, 37.883°, 38.404°, and 38.609°). These results fully indicate that Rho can purify and mineralize solid waste and achieve the purpose of mineral flotation/purification.

**FIGURE 5:**
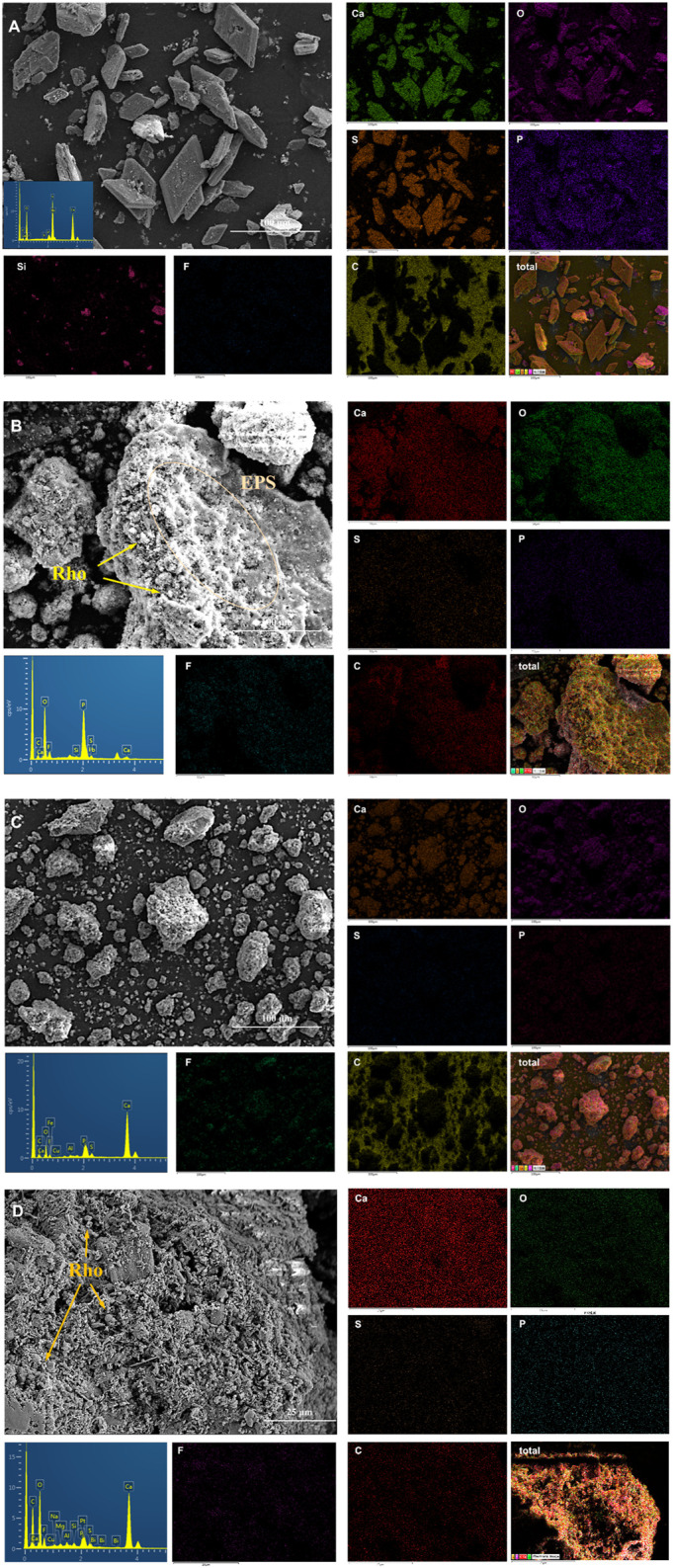
Scanning electron microscope (SEM) and energy-dispersive X-ray (EDX) spectroscopy mapping images of solid after bioleaching by Rho. **(A)**: PG, **(B)**: PG5-Rho, **(C)**: TG, and **(D)**: TG5-Rho. (PG: phosphogypsum; TG: titanium gypsum; Rho: red yeast).

In order to further explore the biological and mineral element characteristics of TG and PG with and without red yeast, four treatments (PG5, TG5, PG5-Rho, and TG5-Rho) were compared and analyzed by SEM. In the SEM diagram, most of PG presents a regular diamond shape with a smooth surface. A small part of PG is broken, but its shape is also very regular (Figure 5A). The culture medium had no obvious adhesion on the surface of PG. EDS results show that S, Ca, P, Si, and other elements have the strongest signals. After Rho bioleaching, obvious Rho cells and many irregular colloidal materials on the surface of PG (Figure 5B) were found. Moreover, it was also found that Rho adhered and wrapped on PG, and caused the morphological changes of PG decomposition and fragmentation (Supplementary Figure S2). These irregular colloidal materials are very similar to extracellular secretions (EPS) of Rho, and their encapsulation of PG is conducive to better adsorption of harmful elements in PG (Figure 5B and Supplementary Figure S2). Meanwhile, the signal of F and P elements appeared near the cells, which further confirmed that Rho could participate in the P transformation and release of PG, and was used to form Ca_3_(PO_4_)_2_ and Ca_5_(PO_4_)_3_F (Figure 5B).

The overall morphology of TG is irregular and granular (Figure 5C). EDS results show that the signals of Ca, O, S, and F in titanium gypsum are very obvious, and the F signal is significantly more than that in PG, which is consistent with the XRF results (Table 1). After bioleaching, Rho cells attached to the surface groove of TG, but there was no obvious colloidal materials, which confirmed that the growth and secretion of Rho were inhibited on TG (Figure 5D). The previous studies have shown that under high stress, red yeast cells themselves will resist the stress of harmful substances in the environment through division and reproduction (Wang et al., 2020), which may also directly affect the production of cell secretions. In TG + Rho, Ca, O, S, and F are still the main elements. Compared with TG5-Rho, P in TG5-Rho is significantly enhanced, and the position of P, F, and S in the figure is highly consistent, which also confirms that Ca_5_(PO_4_)_3_F is the final main mineral. The intervention of red yeast increases the release of F element in PG/TG and promotes it to form stable phosphorus minerals containing F, which is also conducive to reducing the direct harm of F^−^ to the environment after PG/TG application.

### Biomechanism Analysis of Rho for Safe Utilization of PG and TG

**FIGURE 6:**
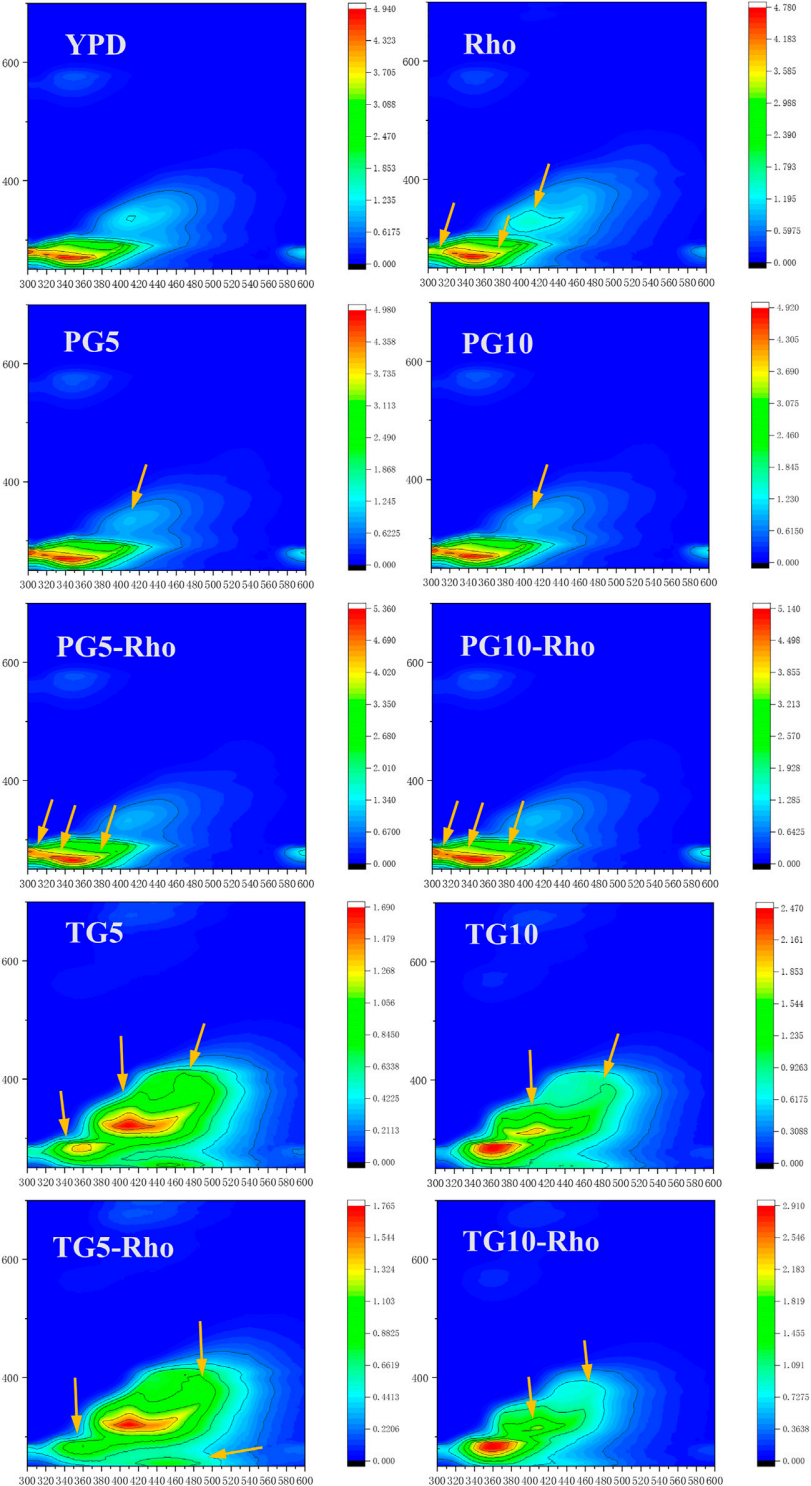
3D-EEM spectra of the reaction solution. (PG: phosphogypsum; TG: titanium gypsum; Rho: red yeast).

Rho can consume organic substances at the Ex/Em 280/310 position in the YPD medium and produce new substances at Ex/Em 350/410, 280/380, and 280/400 positions (Figure 6). The peak position of Ex/Em 350/410 in the PG5/10 medium disappeared, which confirmed that the addition of PG could adsorb the organic components in the culture medium. After Rho addition, the emission wavelength range and intensity of the signal peak at Ex/Em 280/305–320 decrease, which may be due to the reduction of soluble microbial by-product–like material (Chen et al., 2003). Meanwhile, the peak at Ex/Em 280/400 was slightly expanded, which indicates that Rho secretes more organic substances (such as fulvic-like acid substances) (Chen W. et al., 2018). Because Rho grows better in PG5-Rho than in PG10-Rho, the protein content in PG5-Rho was less than that in PG10-Rho.

In TG treatment, the Ex/Em 280/310 peak of YPD disappeared, which confirmed that TG also had adsorption function. Meanwhile, there are large amount of substances with endogenous fluorescence characteristics in TG5/10, such as aromatic hydrocarbon substances (Ex/Em 280/360, 280/400, 320/410, and 380/480). After Rho addition, due to the stress of F^−^ in TG, the peak intensity of Ex/Em at 280/400 was decreased, which means fulvic-like acid substances reduced (Chen W. et al., 2018). In addition, the Ex/Em 280/360, 280/400, 320/410, and 380/480 peaks in YG5-Rho or TG10-Rho were decreased, and the Ex/Em 350/440 peak in TG10-Rho was slightly expanded and the color became lighter. This may be due to the consumption or adsorption of organic matter by microorganisms, which reduces the fluorescent material signal in TG.

**FIGURE 7:**
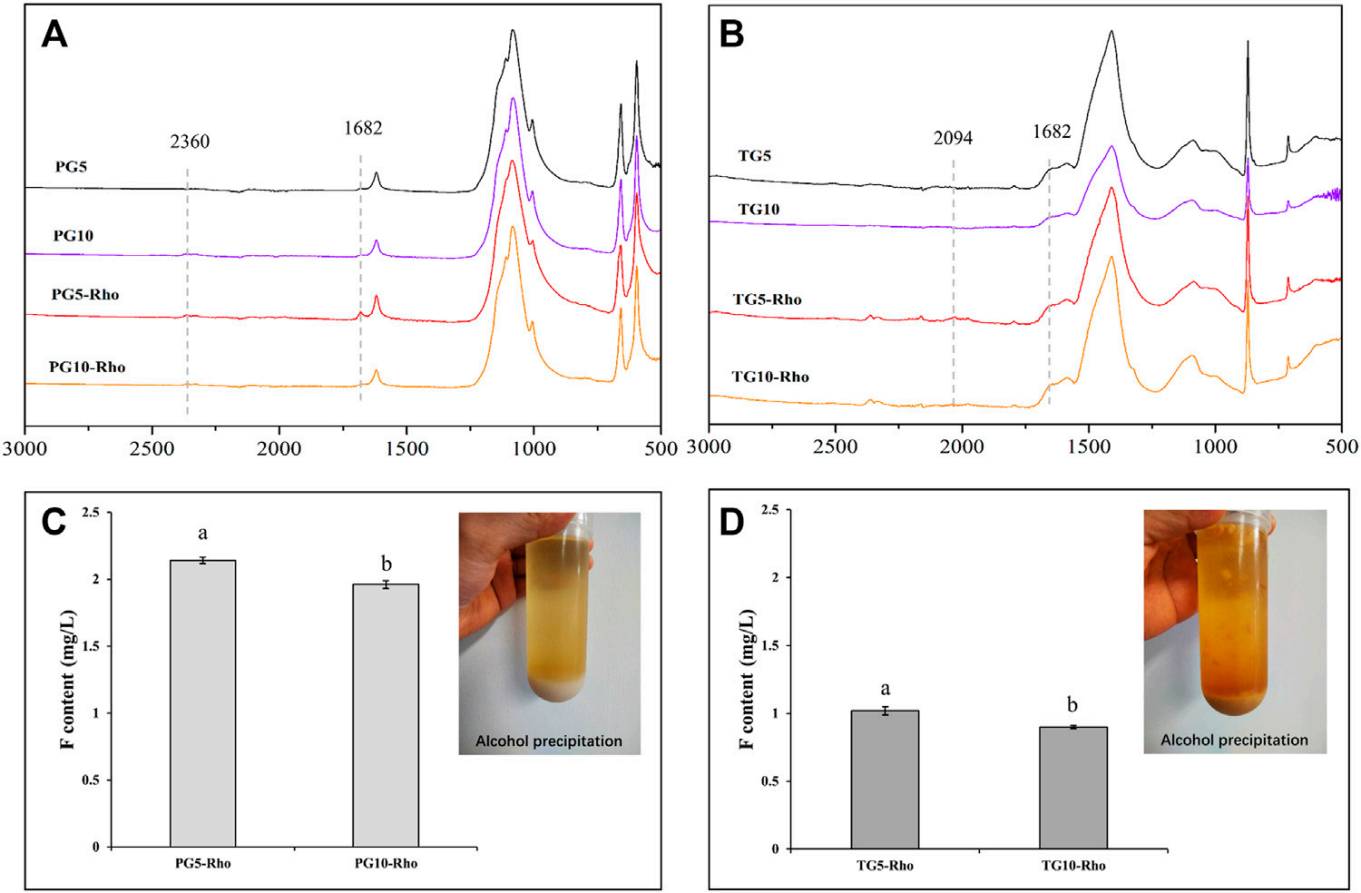
ATR-IR analysis of solid sediment **(A,B)** after centrifugation and content of F in EPS **(C,D)**. (PG: phosphogypsum; TG: titanium gypsum; Rho: red yeast).

The ATR analysis result showed that PG5/10-Rho has obvious EPS peak at 1,682 cm^−1^ (the amide I group C–O stretch) (Maurya et al., 2022), and the peak signal of PG5-Rho is more obvious than that of PG10-Rho (Figures 7A,B), which proves that Rho is more likely to produce organic secretions in PG5. In TG treatment, the peak position of EPS is not obvious, indicating that yeast has less secretion in TG, which may be attributed to the high F stress of TG (Figure 7B). After alcohol extraction, both TG and PG contained obvious EPS (Figure 7B). However, significant Pb, Zn, and Cu (<0.5 mg/L level) were not detected in EPS. This may be because the content of these elements in the raw PG/TG materials is low and cannot be dissolved. Another reason may be that these metal elements are absorbed and fixed by cells. The previous studies also confirmed that yeast cells can adsorb a large amount of metal elements such as Pb and Cu, and can reproduce and grow normally at low heavy mental concentrations (<50 mg/L) (Wang et al., 2020; Wang et al., 2021). Ion chromatography was used to determine the EPS solution (after the extract was dialyzed) and found that there was F ion in the EPS solution, and the content of F in PG was significantly higher than that in TG (Figures 7C,D). Because Rho releases more F in PG than TG (Figure 2D), the adsorption effect of EPS is better. In addition, the content of F in PG10-Rho was 8.4% lower than that in PG5-Rho, and TG10-Rho was 11.8% lower than that in TG10-Rho. In the environment with less solid waste addition, Rho can more easily play its ecological function and accelerate the release of F ions. Combined with the F release of PG5/10 and TG5/10 (Figure 2D), the F adsorption capacity of EPS in PG accounts for 5.3–19.3%, while the F adsorption capacity of EPS in TG accounts for 17.2–29.1%. This result further shows that EPS of Rho has a significant adsorption effect on F in solid waste, which is also one of the main means for Rho to purify PG/TG.

There are many ways of resource utilization of PG, most of which are directly applied to the soil or the beneficial elements (K, Ca, P, *etc*.) are extracted by a chemical replacement method (Sengupta and Dhal, 2021; Yelatontse and Mukhachev, 2021). However, although the cost of soil application is low, the allowable amount of phosphogypsum in soil is limited, and it is easy to cause secondary pollution. The chemical exchange method has the greatest economic benefit, but it is invested too much in the early stage, and the process is complex. For example, the cost of potassium carbonate used for wet conversion of PG by potash solution can reach 1,000–1100 USD/t (Yelatontse and Mukhachev, 2021). However, the cost of using agricultural organic waste (peanut bran, straw, and soybean) for microbial fluid by biological fermentation is only 100–300 USD/t, and that of the agro-industrial effluent medium is even as low as 75–83 USD/t (Martinez-Burgos et al., 2021). The biological flotation/purification technology of high EPS-producing bacteria (red yeast) is simpler and faster. The research also found that the red yeast biological purification technology has the characteristics of easy separation (centrifugation or filtration) and can separate PG/TG and cell bodies at low cost. The purified PG/TG can be recycled and applied to the soil remediation industry, such as passivation and fixation of soil heavy metals, improvement of soil crop fertility, and regulation of soil pH and structure. The Pb or F in yeast cells and secretions can be displaced by chemical purification (acid decomposition), carbonization purification (pyrolysis), and other technologies for resource utilization, so as to greatly reduce the potential harm caused by biological extraction.

## Conclusion

The bioleaching function of red yeast can purify solid waste and reduce harmful elements in mineral solid waste. Reducing red yeast stress environment (mineral solid waste dosage) and increasing yeast biomass are important factors to improve the resource utilization efficiency of mineral solid waste by red yeast. This study found that there are three main mechanisms for the resource/safety utilization of solid waste (PG/TG) by red yeast (Rho) bioleaching. 1) Red yeast can release acidic substances and reduce the environmental pH value, so as to accelerate the dissolution of mineral solids waste and increase the release of harmful elements in mineral. 2) Red yeast can absorb harmful elements (F) in mineral solid waste through its own organisms (cells and EPS), so as to reduce the content of harmful elements in solid and reduce the secondary pollution of harmful elements in the process of bioleaching. 3) Red yeast can promote the mineralization of Ca and F through the production of PO_4_^3−^, so as to make the harmful elements in solid more stable and greatly reduce the environmental risk of solid waste. The content of F is the main factor that determines that the tolerance of red yeast to PG is much higher than that of TG. Red yeast bioleaching can improve the release of P in PG and TG. Because the harmful elements in PG are lower, bioleaching is easier. Therefore, PG is rich in beneficial elements, which makes it easier to be used in soil remediation and improvement.

## Data Availability

The original contributions presented in the study are included in the article/Supplementary Material; further inquiries can be directed to the corresponding authors.
