# Supplementary material for: Red Yeast Improves the Potential Safe Utilization of Solid Waste (Phosphogypsum and Titanogypsum) Through Bioleaching
Source: Front Bioeng Biotechnol. 2021 Dec 31;9:777957. doi: 10.3389/fbioe.2021.777957 (PMC8758580; doi:10.3389/fbioe.2021.777957)
Supplement: Supplementary file 1 [file DataSheet1.docx]

**Supplementary material**


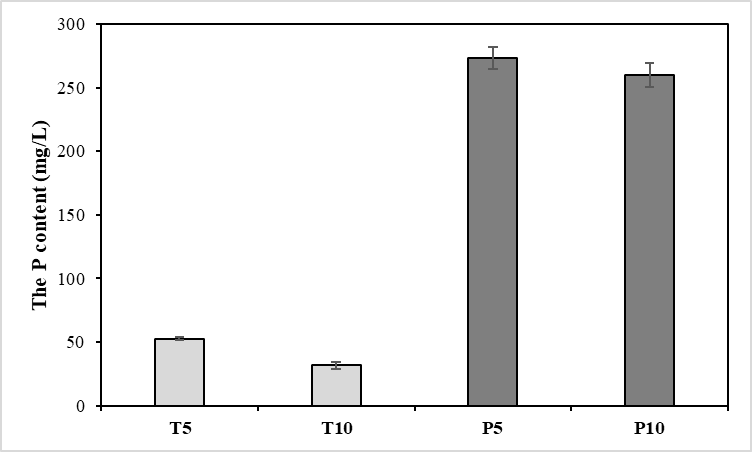


**Figure S1. The P content of Titanium gypsum and Phosphogypsum in deionized water.**


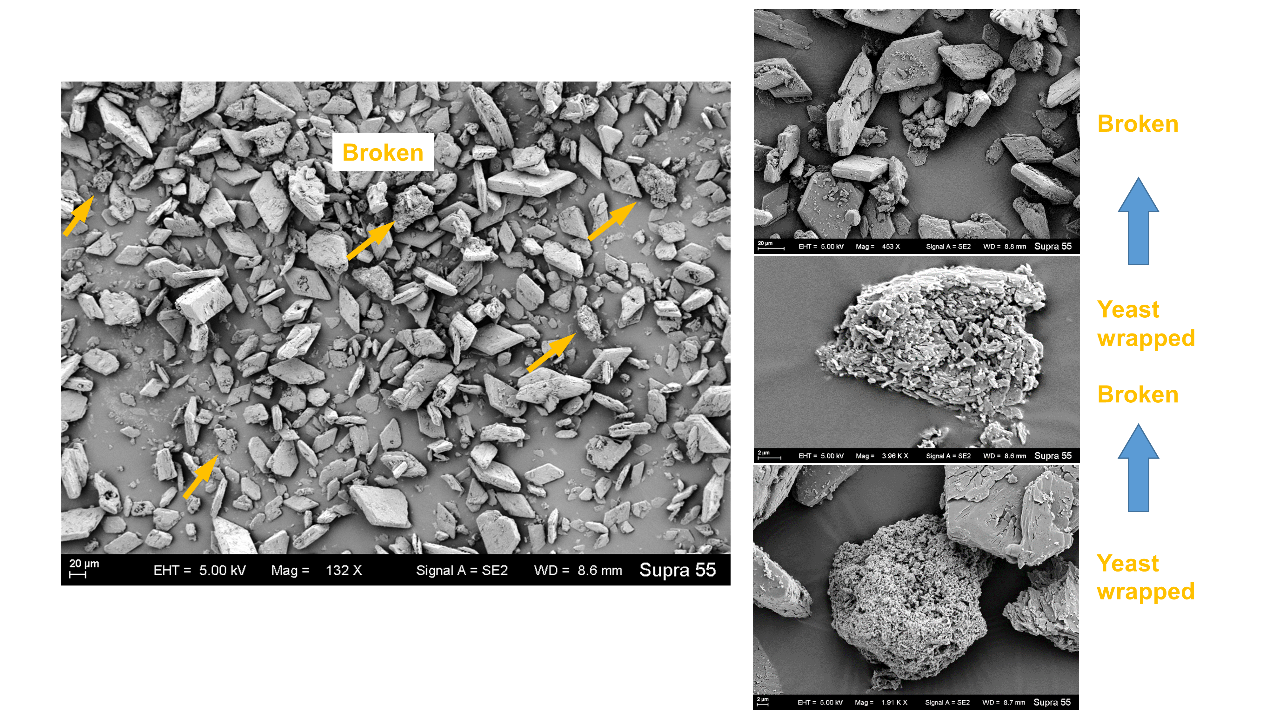


**Figure S2. The adhesion, encapsulation and fragmentation of Red yeast (Rho) to phosphogypsum.**
